# A plasma protein derived TGFβ signature is a prognostic indicator in triple negative breast cancer

**DOI:** 10.1038/s41698-019-0082-5

**Published:** 2019-04-02

**Authors:** Hiroyuki Katayama, Peiling Tsou, Makoto Kobayashi, Michela Capello, Hong Wang, Francisco Esteva, Mary L. Disis, Samir Hanash

**Affiliations:** 1 MD Anderson Cancer Center, 6767 Bertner Avenue, Houston, TX 77030 USA; 2 Division of Hematology/Oncology, Laura and Isaac Perlmutter Cancer Center, New York University Langone Medical Center, New York, NY 10016 USA; 3 Tumor Vaccine Group, University of Washington, 850 Republican Street, Box 358050, Seattle, WA 98109 USA

## Abstract

We investigated the potential of in-depth quantitative plasma proteome analysis to uncover proteins predictive of progression and metastasis in triple negative breast cancer (TNBC). Analysis of samples from 24 pre-menopausal and 24 post-menopausal women with newly diagnosed TNBC who subsequently developed metastasis or remained metastasis free were utilized in the proteomic discovery set, which resulted in 43 proteins associated with tumor progression. These proteins were found to form a hierarchical network with TGFβ. The signature was further confirmed and refined by integrating plasma protein data from a murine TNBC model that encompassed mice with rapid- versus slow-growing tumors. Three genes consisting of CLIC1, MAPRE1, and SERPINA3 in the refined TGFβ signature significantly stratified overall survival (log-rank *p* = 0.0141) in a larger validation cohort irrespective of menopausal status, tumor stage, grade, and size.

## Introduction

Triple negative breast cancer (TNBC) is a heterogeneous subtype of breast cancer that lacks expression of estrogen receptor (ER), progesterone receptor (PR), and human epidermal growth factor receptor 2 (HER2) amplification on the cell surface. Although TNBC comprises a relatively small percentage of breast cancers (10 to 15%), patients with TNBC tend to have a higher risk of both local and distant recurrence, with metastasis more likely to occur in the brain and lungs compared to other subtypes.^1^ The lack of high-frequency oncogenic driver mutations in TNBC limits molecularly targeted therapy options. Currently, chemotherapy remains the main standard of care for patients with TNBC.^2^

Whole-genome sequencing and comprehensive tumor transcriptome profiling have provided information about intrinsic tumor features.^3,4^ Since cancer metastasis involves multi-step processes stemming from interactions between the tumor and various host cell types and extracellular factors, we sought to determine whether plasma proteome profiling may uncover protein signatures for TNBC tumors that are likely to progress.

Transforming growth factor-β (TGFβ) plays a crucial role in promoting tumor progression, including evasion of immune surveillance, autocrine mitogen and cytokine production, epithelial–mesenchymal transition, and myofibroblast and osteoclast mobilization.^5^ Molecular profiling of fast- and slow-growing tumors from the same strain of ER− breast cancer mouse model revealed that transforming growth factor-β (TGFβ) messenger RNA (mRNA) expression was significantly higher in the fast-growing tumor group.^6^ Cells with a high metastatic potential exhibited a higher degree of heat shock factor-1 (HSF1) activation compared to low-metastatic cells established from the same tumor.^7^ Reprogramming of tumor stroma by HSF1 was found to be a potent enabler of malignancy caused by an autocrine TGFβ loop.^8^

We provide in this study evidence for a plasma TGFβ-related protein signature that is predictive of TNBC tumor progression. Expression of genes encompassed in the signature was found to be predictive of survival in an independent TNBC cohort.

## Results

### Differentially expressed plasma proteins between subjects with TNBC who developed metastasis or remained metastasis free

We performed an in-depth quantitative mass spectrometry profiling of plasmas from newly diagnosed TNBC subjects who either subsequently developed metastasis or remained metastasis free (Table 1, Supplementary Fig. S1). The clinical characteristics of four comparison groups matched by stage and menopausal status who provided plasmas are presented in Supplementary Fig. S2. A total of 1618 proteins were quantified in the plasma. First, we carried out a pairwise comparison of plasma proteins between women who developed metastasis (M) versus women who remained free of metastasis (non-M) during the follow-up period within individual groups which yielded four sets of potential progression-related protein candidates for the four groups (supplementary Table S1). Differentially expressed proteins between M and non-M in the four groups shared common biological processes, including immune related, wound healing, and cell motility based on gene ontology enrichment analysis (supplementary Table S2). TGFβ1 and tumor necrosis factor (TNF) ranked as the top regulators in Upstream Regulator Analysis using Ingenuity Pathway Analysis®^9^ (www.ingenuity.com) (supplementary Table S3).

**Table 1:** Clinical characteristics of TNBC patients of the current study

			Age at diagnosis			Duration of follow-up (diagnosis to endpoint)		
Menopause status	Stage		Non-M	M		Non-M	M	
			(*n* = 9/group)	(*n* = 3/group)	*P*	(*n* = 9/group)	(*n* = 3/group)	*P*
Pre	II	Mean ± SD	42.7 ± 3.2	46.0 ± 5.4	0.26	3.7 ± 2.0	2.3 ± 1.3	0.32
		Median ± SD	41.0 ± 3.2	45.0 ± 5.4		3.3 ± 2.0	1.7 ± 1.3	
	III	Mean ± SD	38.2 ± 6.9	36.7 ± 3.7	0.73	4.0 ± 0.9	1.0 ± 0.3	<0.001
		Median ± SD	38.0 ± 6.9	37.0 ± 3.7		4.4 ± 0.9	0.9 ± 0.3	
Post	II	Mean ± SD	63.4 ± 7.0	60.3 ± 5.3	0.54	4.6 ± 2.3	3.3 ± 0.4	0.37
		Median ± SD	64.0 ± 7.0	60.0 ± 5.3		3.7 ± 2.3	3.4 ± 0.4	
	III	Mean ± SD	57.6 ± 5.4	52.3 ± 6.6	0.24	3.4 ± 1.6	1.9 ± 1.1	0.20
		Median ± SD	58.0 ± 5.4	56.6 ± 6.6		3.4 ± 1.6	2.0 ± 1.1	

*TNBC* triple negative breast cancer, *M* metastatic, *non-M* non-metastatic

**Fig. 1:**
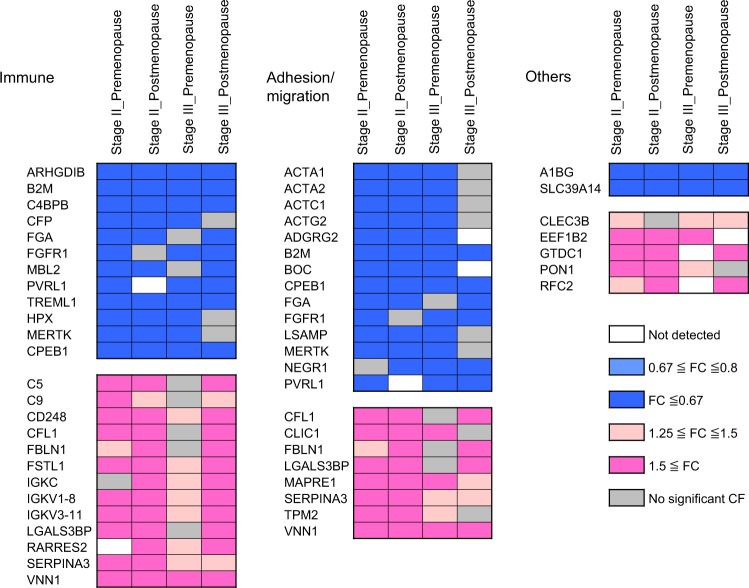
Immune and cell adhesion/migration are top two enriched biological processes for progression-related proteins. The 43 progression-related plasma proteins were subjected for enrichment analysis with Gene Ontology (GO) annotation implemented in the STRING (Search Tool for the Retrieval of Interacting Genes/Proteins) database. Top two enriched biological processes were immune (left) and cell adhesion/migration (middle). Eleven proteins were annotated in both immune cell and adhesion/migration. The fold change (FC) of 43 progression-related proteins between metastasis (M) and non-metastasis (non-M) from the 4 triple negative breast cancer (TNBC) cohorts are depicted using the color scale shown above

In total, 22 and 21 plasma proteins exhibited concordant and significantly increased or decreased, respectively, in all four groups. Enrichment analysis with Gene Ontology (GO) annotation was implemented using the STRING (Search Tool for the Retrieval of Interacting Genes/Proteins) database^10^ and included functional annotation from Uniprot,^11^ Entrez Gene (https://www.ncbi.nlm.nih.gov/gene/), and PubMed searches (supplementary Table S4). A total of 24 of the 43 proteins were designated as extracellular (GO: 00055576), whereas 21 and 19 proteins were annotated as membrane-bound vesicle (GO: 0031988) or extracellular exosome related (GO: 0070062), respectively (Supplementary Table S4). The most enriched biological process was humoral immune response which included 25 proteins. The second most enriched biological process was cell adhesion/migration (Fig. 1, Table 2, and supplementary Table S4). Eleven proteins were annotated in both immune cell and adhesion/migration, consisting of B2M, CPEB1, FGA, FGFR1, MERTK, PVRL1 (NECTIN1), CFL1, FBLN1, LGALS3BP, SERPINA3, and VNN1.

**Table 2:** The numbers of enriched GO terms and the containing plasma proteins

		Enriched GO terms^a^	Differential plasma proteins^a^	Range of FDR
Immune		18	25	5.55E−05–4.87E−02
Immune system process		3	14	3.28E−04–4.23E−02
Immune system regulation		5	12	1.17E−03–4.17E−02
Innate immunity		1	11	5.12E−04
Humoral immunity		3	7	4.76E−05–5.12E−04
Complement		4	5	5.55E−05–1.50E−03
Defense		2	14	1.59E−04–4.87E−02
Curated from literature		NA	6	NA
Adhesion/migration		6	22	4.31E−07–4.23E−02
Adhesion		3	9	8.90E−03–4.23E−02
Mesenchymal migration		3	6	4.31E−07–7.62E−04
Curated from literature		NA	7	NA

^a^More specific terms were merged into a broader module as summarized above (see supplementary Tables 1, 2 for a complete list of all gene ontology enriched terms)

*GO* gene ontology, *FDR* false discovery rate, *NA* not available

### Progression-related plasma proteins form a TGFβ-regulated network

**Fig. 2:**
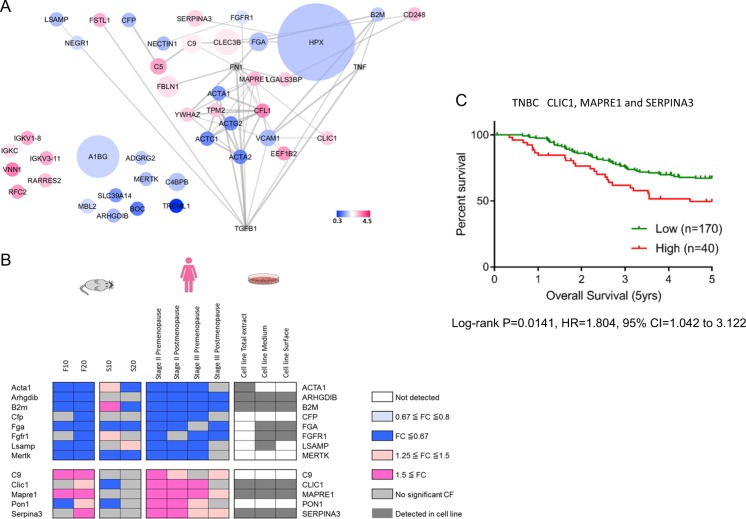
Transforming growth factor-β (TGFβ) signature identified from triple negative breast cancer (TNBC) pre-metastatic vs non-metastatic plasmas. **a** A hierarchical network regulated by TGFβ was formed by progression-related proteins. The size of the nodes represents the relative abundance (not in linear scale) of the plasma protein, while the color represents the fold changes (metastasis (M) vs non-metastasis (non-M)) as the color scale shown above. Gray nodes represent edited hub proteins (see text). **b** A refined signature was generated by integrating data from plasma of slow (S) vs fast (F) progressor mice and human TNBC cell lines proteome. F10 and S10 represent fold changes of plasma protein at first time point (far from diagnosis, see Methods) relative to baseline time point. F20 and S20 represent fold changes of plasma protein at second time point (closer to diagnosis, see Methods) relative to baseline time point. The magnitude of fold change is shown as the color scale above. CFP, C9, and PON1 were not detected in TNBC cell lines and thus were removed from the “tumor-intrinsic” signature. **c** The plasma-derived signature composed of three proteins (CLIC1, MAPRE1, and SERPINA3) that were higher in the metastatic group was evaluated in the independent human TNBC cohort

To further explore the relations among plasma proteins associated with metastasis, we first queried the protein–protein interaction (PPI) database. Of the original 43 progression-related plasma proteins, 40 mapped in the STRING database^10^ and yielded a sparse network with 3 small cliques (supplementary Fig. S3). The top three hub proteins, FN1, VCAM1, and YWHAZ, were selected for further analysis (see Methods for selection criteria). The top two upstream regulators, TGFβ and TNF, were also included in the analysis (supplementary Table S3). The overall topology of the network was analyzed using the network analyzer in Cytoscape. The clustering coefficient, average number of neighbors, and characteristic path length were 0.145, 2.444, and 2.920, respectively. All nodes were placed in hierarchically arranged layers using yFiles layouts (yWorks®) in Cytoscape.^12^ Interestingly, this generated a layered structured interconnected network with TGFβ as a key regulator (Fig. 2a).

### Concordance of plasma proteins associated with progression between human and a TNBC mouse model

We investigated a well-established murine TNBC model, C3(1)-Tag mice, that develops tumors which exhibit a similar gene expression pattern and histopathological characteristics as human basal-like breast cancers.^13^ Mice in this model exhibit a dichotomous pattern of tumor progression.^6^ We harvested plasma at baseline and at two additional pre-clinical time points from mice bearing slow- versus fast-growing tumors which resulted in 13 proteins in common between both human and mouse cohorts (Fig. 2b, supplementary Fig. S4). Of the 13 proteins (Fig. 2b, supplementary Table S6, S7), 9 were found to be expressed in 17 TNBC cell lines, and therefore were considered to be potentially contributed to by tumor cells.

The association of these 9 proteins with TGFβ was further examined. Five proteins, including ACTA1, B2M, FGA, FGFR1, and SERPINA3, were known to be regulated by TGFβ based on published reports^14–20^ and on IPA upstream analysis.^9^ For ARHGDIB, CLIC1, LSAMP, and MAPRE1, their connection with TGFβ has not been previously reported. Further analysis of data from The Cancer Genome Atlas (TCGA) and The Clinical Proteomic Tumor Analysis Consortium (CPTAC) provided evidence for the association of these proteins with TGFβ. Eight of these nine proteins, aside from FGFR1, are significantly correlated with TGFβ either from mRNA or protein data or both (supplementary Table S8).

Among the 17 TNBC cell lines referenced to the plasma data sets, we characterized the secretome of two metastatic (BPLERs) and two non-metastatic (HMLERs) cell lines^7^ to identify proteins in the extracellular space that could be related to metastasis. Analysis of media for differentially expressed proteins in BPLER3 vs HMLER3 and BPLER2 vs HMLER2 resulted in 443 proteins that were commonly elevated in BPLER (BPLER/HMLER>=1.5) and 319 proteins that were commonly down-regulated (BPLER/HMLER<=0.67). The Upstream Regulator of Ingenuity Pathway Analysis confirmed the significance of TGFβ as a major network and predicted the activation of TGFβ network (supplementary Table S9, Fig. S5). Twelve differentially expressed proteins from the BPLER and HMLER media compartment overlapped with the 43 plasma TGFβ signature proteins related to progression (supplementary Table S9), providing further support for a TGFβ signature in plasma associated with metastasis.

### Expression of TGFB signature genes stratifies TNBC patients

We next determined whether the three genes CLIC1, MAPRE1 and SEPINA3 that were concordantly upregulated in human metastatic group and mouse fast-growing tumor group can stratify overall survival of TNBC patients using the dataset of Curtis et al.^21^ The survival curve based on the mRNA levels of signature genes was significantly prognostic in 210 TNBC patients cohort (log rank *p* = 0.0141, hazard ratio (HR) = 1.804, 95% confidence interval (CI) = 1.042–3.122) described in Fig. 2c. Taking stage, menopause status, tumor grade, and tumor size into consideration, we further performed analysis using uni- and multi-variate Cox regression model in TNBC dataset. The three gene panel exhibited significance as an independent risk factor in both analyses (Table 3).

**Table 3:** Uni- and multi-variate Cox proportional hazards models of TNBC cohort

		β	HR	95% CI	*P*
Univariate analysis					
Gene panel (CLIC1, MAPRE1, SERPINA3)					
	Low vs high	0.50072	1.64992	1.10339–2.46715	0.0147
Stage					
	0–1 vs 2–4	0.09793	1.10288	0.76324–1.59366	0.6021
Menopause					
	Pre vs post	0.2572	1.29331	0.89125–1.87673	0.1758
Tumor grade					
	1–2 vs 3	0.14607	1.15728	0.65995–2.02939	0.6102
Tumor size					
	<31 mm vs 31 mm ≦	0.33134	1.39284	0.93430–2.07642	0.1039
Multivariate analysis					
Gene panel (CLIC1, MAPRE1, SERPINA3)					
	Low vs high	0.55067	1.73442	1.15555–2.60327	0.0079
Stage					
	0–1 vs 2–4	0.39731	1.48782	0.99404–2.22689	0.0535
Menopause					
	Pre vs post	n/d	n/d	n/d	n/d
Tumor grade					
	1–2 vs 3	n/d	n/d	n/d	n/d
Tumor size					
	<31 mm vs 31 mm ≦	n/d	n/d	n/d	n/d

*TNBC* triple negative breast cancer, *HR* hazard ratio, *CI* confidence interval, *n/d* not detected

## Discussion

The role of TGFβ in tumor progression is well established at the tissue level.^5,22^ Our study uncovered a TGFβ plasma protein signature predictive of tumor progression based on interaction network analysis^23^ using established public resources which contain both known and predicted PPIs as in the case of the STRING database.^10^ Despite extensive curation efforts, the existing maps are considered incomplete,^24^ we thus also included manually curated interactions from the literature.

While the relationship between TGFβ and cancer progression has been well appreciated,^5,22^ uncovering a network associated with this critical driver in TNBC plasma predictive of metastasis as an independent prognostic indicator is novel. The commonly observed TGFβ pathway signature genes are mainly transcriptional targets downstream of SMADs,^5,25^ whereas the connection of our plasma proteins with TGFβ is based on PPIs. Thus, the make-up of the signature is different from what has been previously reported. Nonetheless, the expression level of genes corresponding to most proteins with differential expression in plasma between the metastatic and the non-metastatic groups (33 out of 39 progression-related proteins and 8 of the 9 signature proteins) correlated with TGFβ at either mRNA, protein, or both. Interestingly, the TGFβ network we uncovered exhibited characteristics of a scale-free network: with the degrees of nodes following a power law distribution. In contrast to a random network, accidental loss of individual non-hub vertices in our network would be less disruptive,^26^ suggesting that the topology of our network is quite robust. The occurrence of proteins with either increased or decreased levels in the TGFβ signature (Fig. 2a) may be due to an added autoimmune response resulting in protein binding as part of immune complexes with increased clearance for some of the proteins. Analysis of plasma immunoglobulin G (IgG)-bound proteins by mass spectrometry separately from free circulating proteins identified ACTG2, FGA, FGFR1, HPX, and TNF that were decreased in the metastasized cohort as bound to IgG.

The network approach not only provided biologically meaningful insights on a global view but also revealed novel proteins, including CLIC1 (chloride intracellular channel 1), LSAMP (limbic system-associated membrane protein), and MAPRE1 (microtubule-associated protein RP/EB family member 1) that were not previously linked to the TGFβ network. The minimal step size for CLIC1, LSAMP, and MAPRE1 to TGFβ is 2; i.e., taking into consideration the association of one additional progression-related plasma protein from our dataset links these proteins to TGFβ (Fig. 2a, Supplementary Table S8A). LSAMP encodes a preproprotein that is proteolytically processed to a neuronal surface glycoprotein, acting as a selective homophilic adhesion molecule for axon guidance and neuronal growth in the developing limbic system. It has been reported as candidate tumor suppressor in human osteosarcomas^27^ and clear cell renal carcinomas.^28^ MAPRE1 is reported as a plasma biomarker for early-stage colorectal cancer and adenoma.^29^ CLIC1 is connected to VCAM1 and FN1, an essential component of the extracellular matrix (Supplementary Table S8). It plays a critical role in the stability of invadopodia in endothelial and tumor cells and the regulation of cell–extracellular matrix interactions and ability of tumor cells to metastasize to distant organs.^30^ Additionally, CLIC1 has been proposed as a novel prognostic marker for intraperitoneal metastasis in serous epithelial ovarian cancer.^31^ Considering the candidates of predictive TNBC progression marker showed higher in metastasis, SERPINA3 was grouped together in addition to CLIC1 and MAPRE1 (Fig. 2b, supplementary Fig S7). SERPINA3 in colon cancer tissue was significantly elevated and associated with patient’s pathological features, and knocking down the gene in colon cancer cell lines decreased migration and invasiveness of the cells which resulted in reducing the liver metastasis in xenograft model.^32^ The three genes CLIC1, MAPRE1 and SERPINA3 represented by the refined TGFβ signature significantly stratified overall survival in independent human cohort which was specific to TNBC (Fig. 2c, Table 2).

In the metastatic BPLER cells induced by HSF1 activation, secretome analysis uncovered TGFβ as significantly elevated in comparison with non-metastatic HMLER in the Ingenuity Upstream Regulator Analysis. TGFβ-HSF1 was also found to be a key factor of CAF development and metastasis in TNBC.^8^ Another setting of TGFβ-regulated metastasis observed in TNBC was found to be driven by serglycin (SRGN) induction stimulated by TGFβ via autocrine and paracrine loops.^33^ Although the master regulators HSF1 and SRGN were different in these two settings, TGFβ was involved in TNBC metastasis likely through an effect on the tumor microenvironment which was reflected in a TGFβ network in plasma.

The three-marker panel of CLIC1, MAPRE1 and SERPINA3 exhibited significance regardless of tumor stage, grade, size, and menopausal status. In our cohort, subjects in the metastatic group with high values for our marker panel recurred within 1.0–3.3 years from diagnosis, whereas subjects with low values in non-metastatic group were recurrence free during the 3.4–4.6 years of follow-up. Similarly, the marker panel predicted overall survival in the larger cohort validation set. Our findings justify further validation in prospective studies.

## Methods

### Plasma sample collection and processing

#### Human TNBC cohorts

Plasma samples were collected from women with newly diagnosed (0–0.8 years) TNBC. Only stage II and stage III patients who had no documented distant metastasis at the time of sample collection were included in this study. Written informed consent was obtained and the study was approved by the institutional review board at MD Anderson Cancer Center. The timing of blood draw was after the diagnostic biopsy and prior to neoadjuvant chemotherapy, or definitive surgery in patients who did not receive chemotherapy in the neoadjuvant setting. Anonymized individual patient information is presented in supplementary Table S10, Clinical data.

For mass spectrometry profiling, we compiled four cohorts with matched age, stage, and menopausal status. In each cohort, 9 women diagnosed with TNBC who did not metastasize during follow-up was defined as “non-metastasized” (non-M), while 3 pre-menopausal and 3 post-menopausal women who metastasized in the follow-up period was defined “metastasized” (M). The follow-up was started at the time of diagnosis in both non-M and M. The patients were determined to be metastasis free at the time of presentation by chest X-ray, bone scan, and computed tomography of the abdomen. A complete blood cell count and chemistry panel including liver function tests, kidney function, lactate dehydrogenase, and alkaline phosphatase were all within normal limits at the time of presentation. Serum markers were not drawn because patients had early-stage breast cancer at the time of blood collection, as recommended by the ASCO (American Society for Clinical Oncology) guidelines. As shown in supplementary Fig. S2 and Table 4, these four cohorts were comparable. Details of each patient information is listed in Supplementary Table S10. Individual patient plasmas were pooled to total of 100 µL in each sample group for the analysis.

**Table 4:** Chemotherapy treatments of the TNBC patients

	Non-M		M	
	(*n* = 36)	(%)	(*n* = 12)	(%)
TNM-M0 at the time of diagnosis	36	100.0	12	100.0
No chemotherapy	3	8.3	0.0	0.0
Chemotherapy	33	91.7	12.0	100.0
Neoadjuvant chemotherapy	26	72.2	10.0	83.3
Adjuvant chemotherapy	7	19.4	2.0	16.7
Chemotherapy regimens				
Taxane	32	88.9	12.0	100.0
Anthracycline	33	91.7	12.0	100.0
Cyclophosphamide	33	91.7	12.0	100.0

*TNBC* triple negative breast cancer, *TNM* tumor, node, metastasis, *M* metastatic, *non-M* non-metastatic

### Slow versus fast progressor TNBC mouse model

Breast cancer mouse model of C3(1)-Tag was used as basal type. Slow tumor-growing group confirmed tumor in average 20 weeks and the plasma of baseline, prediagnostic 1, and prediagnostic 2 were collected at 7, 16, and 19 weeks, respectively. Fast tumor group confirmed tumor in average 16 weeks and the plasma of baseline, prediagnostic 1, and prediagnostic 2 were collected at 6, 11, and 13 weeks, respectively. Four individual mice in each time point were pooled to total 60 µL for the analysis. All procedures were done in accordance with the University of Washington Institutional Animal Care and Use Committee guidelines.

### Depletion of abundant proteins

A total of 60 µL of pooled mouse plasma for each experimental condition was processed with the immune-depletion column Mu-3 10 × 100 mm (Agilent Technologies, #5188–5218) to remove top-3 high abundance proteins, Albumin, IgG, and Transferrin.

For human plasma, a total of 100 µL of pooled sample for each experimental condition was processed with the immuno-depletion column Hu-14 10 × 100 mm (Agilent Technologies, #5188–6559) to remove top-14 high abundance proteins, Albumin, IgG, IgA, Transferrin, Haptoglobin, Fibrinogen, α1-Antitrypsin, α1-Acid Glycoprotein, Apolipoprotein AI, Apolipoprotein AII, Complement C3, Transthyretin, IgM, and α2-Marcroglobulin. The flow-through fraction was used for lower abundance plasma free proteome.

### Protein labeling and fractionation prior to mass spectrometry

The immuno-depleted flow-through fraction was concentrated using concentrator (Ultracel-3k, 3k molecular weight (MW) cut/off, Merck) in centrifuge 4000 × *g* at 4 °C and reduced with 25 mM TCEP (tris(2-carboxyethyl)phosphine). The reduced Cys was labeled with 6 plex Iodoacetyl tandem Mass Tag (Thermo Scientific, IodoTMT #90102). Next, 50 µl of methanol was added to 0.2 mg TMT vial to dissolve the reagent, mixed and centrifuged at 1000 × *g* for 1 min. The Cys reduced protein sample was added to TMT vial, centrifuged at 1000 × *g* for 1 min, and the TMT labeling reaction was left at 37 °C for 1 h in dark. Then, the TMT labeling reaction was quenched by adding 4 µl of dithiothreitol and the sample was incubated for 15 min at 37 °C in dark. The TMT labeled sample was further processed with the buffer exchange using Zeba Column (7k MW cut/off, Thermo Scientific, #89893).

The desalted samples were processed through Shimadzu 2D-HPLC system to fractionate in protein level. The first dimension was anion-exchange chromatography mode (anion-exchange column, 7.5 × 150 mm, Column Technology Inc, #NA75150WP). The sample was fractionated into 8 fractions with the B pump step elution using the mobile phase A (20 mmol/L Tris, 4 mol/L urea, 3% isopropanol) and mobile phase B (20 mmol/L Tris, 4 mol/L urea, 3% isopropanol, 1 mol/L NaCl). The 8 AEX fractions were further separated with the same reversed-phase mode described in IgG-bound analysis and total 96 fractions were obtained. The samples were lyophilized and digested by trypsin for liquid chromatography–mass spectrometry (LC-MS) analysis.

### Liquid Chromatography-High Definition Mass Spectrometry with Expression (LC-HDMS^E^) data acquisition

LC-HDMS^E^ data were acquired in resolution mode with SYNAPT G2-Si using Waters Masslynx (version 4.1, SCN 851). The capillary voltage was set to 2.80 kV, sampling cone voltage to 30 V, source offset to 30 V, and source temperature to 100 °C. Mobility utilized high-purity N2 as the drift gas in the ion-mobility spectrometry (IMS) TriWave cell. Pressures in the helium cell, Trap cell, IMS TriWave cell, and Transfer cell were 4.50 mbar, 2.47e−2 mbar, 2.90 mbar, and 2.53e−3 mbar, respectively. IMS wave velocity was 600 m/s, helium cell DC was 50 V, Trap DC bias was 45 V, IMS TriWave DC bias was 3 V, and IMS wave delay was 1000 μs. The mass spectrometer was operated in V-mode with a typical resolving power of at least 20,000. All analyses were performed using positive mode electrospray ionization (ESI) using a NanoLockSpray source. The lock mass channel was sampled every 60 s. The mass spectrometer was calibrated with a [Glu1] fibrinopeptide solution (300 fmol/µL) delivered through the reference sprayer of the NanoLockSpray source. Accurate mass LC-HDMS^E^ data were collected in an alternating, low energy (MS) and high energy (MSE) mode of acquisition with mass scan range from *m/z* 50 to 1800. The spectral acquisition time in each mode was 1.0 s with a 0.1-s inter-scan delay. In low energy HDMS mode, data were collected at constant collision energy of 2 eV in both Trap cell and Transfer cell. In high energy HDMS^E^ mode, the collision energy was ramped from 25 to 55 eV in the Transfer cell only. The radio frequency applied to the quadrupole mass analyzer was adjusted such that ions from *m/z* 300 to 2000 were efficiently transmitted, ensuring that any ions observed in the LC-HDMS^E^ data less than *m/z* 300 were known to arise from dissociations in the Transfer collision cell. The acquired LC-HDMS^E^ data were processed and searched against protein knowledge database (Uniprot) through ProteinLynx Global Server (PLGS, Waters Company) with false discovery rate of 4%.

### Cell line analysis

A total, 17 breast cancer cell lines HCC1937, HCC1599, HCC1806, MDA-MB-468, HCC70, HCC1187, HS578T, BT549, HCC1395, HCC38, MDA-MB-436, BT20, and MDA-MB-157 were obtained from ATCC, and HMLER2, HMLER3, BPLER2, and BPLER3 were obtained from Dr. Susan Lindquist’s group, Department of Biology, Massachusetts Institute of Technology. Detailed methods for cell culture, collection of total cell extracts, conditioned media, and cell surface proteins and trypsin digestion have been described in ref. ^31^

The trypsin digested peptides of each cell compartment was separated by reversed-phase chromatography using EASYnano 1000 HPLC system (Thermo Scientific) coupled online with a LTQ-Orbitrap ELITE mass spectrometer (Thermo Scientific). Mass spectrometer parameters were spray voltage 2.5 kV, capillary temperature 300 °C, Fourier transform (FT) resolution 60,000, FT target value 1 × 10^6^, LTQ target value 3 × 10^4^, 1 FT microscan with 500 ms injection time, and 1 LTQ microscan with 10 ms injection time. Mass spectra were acquired in a data-dependent mode with the *m/z* range of 350–2000. The full mass spectrum (MS scan) was acquired by the FT and tandem mass spectrum (MS/MS scan) was acquired by the LTQ with a 35% normalized collision energy. Acquisition of each full mass spectrum was followed by the acquisition of MS/MS spectra for the 20 most intense +2 or +3 ions within a 1-s duty cycle. The minimum signal threshold (counts) for a precursor occurring during a MS scan was set at 5000 for triggering a MS/MS scan.

The acquired LC-MS/MS data were processed by the Tans-Proteomic Pipeline (TPP) 4.8. Briefly, LC-MS/MS data were first converted to mzXML format using ProteoWizard to generate the peak list for the protein database searching. The X!Tandem search engine parameters included SILAC 13C6 labeled Lys (6.020129@K) and cysteine (Cys) alkylated with acrylamide (71.03714@C) as a fixed modification and methionine (Met) oxidation (15.99491@M) as a variable modification. Data were searched against the protein knowledge database (Uniprot). The minimum criterion for peptide matching was a Peptide Prophet Score ≥0.2. Peptides meeting this criterion were grouped to protein sequences using the Protein Prophet algorithm at an error rate of ≤5%. Total mass spectrometry counts for each protein was used as a measure of protein abundance.

### Progression-related protein candidates in human and mouse cohorts

The following criteria were applied for the identification of differentially expressed proteins within each individual TNBC cohort. First, for proteins mapped by multiple peptide sequences in the cohort, a paired *t*-test was used to select proteins differentially expressed between M and non-M plasma samples (*p* value < 0.05); for proteins mapped by single peptide, we retained only peptides with higher confidence mapping by removing peptides with multiple protein hits in the protein knowledge database (Uniprot) as well as the ones with lower than 6.0 peptide score (natural log of the likelihood that the fragment spectrum given peptide sequence). Second, fold change between M and non-M must be greater than 1.25 to be selected as candidates. Next, to identify robust progression-related proteins shared by the four cohorts, we carried out a paired sample Student’s *t*-test on protein-level logarithmically loess-normalized data of M vs non-M samples and 22 and 21 plasma proteins were determined to be increased or decreased, respectively, using 1.25-fold change as a cutoff in at least 3 out of 4 cohorts, and paired *t*-test *p* values < 0.1.

For mice, F10 and S10 represent fold changes of plasma protein at prediagnostic 1 (far from diagnosis) relative to baseline time point, and F20 and S20 represent fold changes of plasma protein at prediagnostic 2 (closer to diagnosis) relative to baseline time point. The progression-related proteins were defined with the cutoff of fold change greater than 1.3 for F20.

### Protein-protein interaction network construction and visualization

A PPI network from our progression-related plasma proteins was constructed. Out of the original 43 progression-related plasma proteins, 40 mapped in the STRING database^10^ except for 3 immunoglobulins (IGKC, IGKV1-8, andIGKV3-11) and yielded a sparse network with 3 small cliques connected by 17 edges and 24 isolated nodes. To improve the inter-connectivity of the network, we added 3 hub proteins FN1, VCAM1, and YWHAZ by the following approach. First, we systemically searched the interacting partners of all 40 nodes in 2 pools: 261 plasma proteins that were detected in all 4 cohorts or 97 proteins with fold changes greater than 1.25 in at least 3 out of 4 cohorts. On a combined protein interaction network from the NCBI (National Center for Biotechnology Information) gene database and the STRING database with confidence score greater than 500, the top 2 hits were ALB (9 links) and FN1 (8 links) for first pool and VCAM1 (5 links) and YWHAZ (3 links) for the second. Three hub proteins (except for albumin) were thus selected. The ratios between M and non-M of these three hub proteins are listed in supplementary Table S5. Top two upstream regulators, TGFβ and TNF, were also included (supplementary Table S3). The overall topology of the network was analyzed using the network analyzer in Cytoscape.^12^ To visualize the potential connections of these plasma proteins, all the nodes were placed in hierarchically arranged layers using yFiles layouts (yworks®) in Cytoscape.

### Assessment of prognostic significance of plasma protein-derived signature based on tissue mRNA expression

The prognostic value of our metastasis plasma protein signatures on the BC tissue mRNA expression cohort^21^ was evaluated in the following steps. First, we categorized TNBC and then we defined an overall risk score as the average mRNA level of the three proteins (CLIC1, MAPRE1, and SERPINA3) elevated in metastatic groups. Second, Kaplan–Meier curves were constructed on high- and low-risk groups defined as samples with the upper quarter as a High Risk (*n* = 40) and lower third risk scores as a Low Risk (*n* = 170). HRs were derived from the Cox proportional hazards model, and statistical significance was assessed by log-rank test. Third, uni- and multi-variate Cox proportional hazards models were used to test the effect of risk scores alone or in combination of other clinical variables including age, stage, menopause status, tumor grade, and tumor size. The analysis was performed using StatFlex software (Artech).

### TGFβ association with 43 progression-related proteins

The mRNA and protein expression values for TGFB1 and 43 progression-related proteins were obtained from TCGA Genome Data Analysis Center and CPTAC. Basal type BRCA (based on PAM50 panel) samples were selected. Out of 43 proteins, 39 (except for 3 immunoglobulins and GDGRP2) had mRNA expression data and 32 had protein expression data available. Pearson’s correlation statistics were calculated between TGFB1 and these progression-related proteins on mRNA or protein, respectively.

### Reporting Summary

Further information on experimental design is available in the Nature Research Reporting Summary linked to this article.

## Supplementary information

Reporting Summary

Supplementary Figures

Supplementary Table

## Data Availability

All the data generated or analyzed during this study are included in this article and its supplementary information files or available from the author upon reasonable request.

## Supplementary information

**Supplementary Information** accompanies the paper on the *npj Precision Oncology* website (10.1038/s41698-019-0082-5).

## References

[1] Hurvitz S, Mead M (2016). Triple-negative breast cancer: advancements in characterization and treatment approach. Curr. Opin. Obstet. Gynecol..

[2] Bianchini G, Balko JM, Mayer IA, Sanders ME, Gianni L (2016). Triple-negative breast cancer: challenges and opportunities of a heterogeneous disease. Nat. Rev. Clin. Oncol..

[3] Cancer Genome Atlas Research Network. (2012). Comprehensive molecular portraits of human breast tumors. Nature.

[4] Ciriello G (2015). Comprehensive molecular portraits of invasive lobular breast. Cancer Cell.

[5] Padua D, Massague J (2009). Roles of TGFbeta in metastasis. Cell Res..

[6] Gad E (2014). Natural history of tumor growth and immune modulation in common spontaneous murine mammary tumor models. Breast Cancer Res. Treat..

[7] Mendillo ML (2012). HSF1 drives a transcriptional program distinct from heat shock to support highly malignant human cancers. Cell.

[8] Scherz-Shouval R (2014). The reprogramming of tumor stroma by HSF1 is a potent enabler of malignancy. Cell.

[9] Kramer A, Green J, Pollard J, Tugendreich S (2014). Causal analysis approaches in Ingenuity Pathway Analysis. Bioinformatics.

[10] Szklarczyk D (2017). The STRING database in 2017: quality-controlled protein-protein association networks, made broadly accessible. Nucleic Acids Res..

[11] UniProt C (2015). UniProt: a hub for protein information. Nucleic Acids Res..

[12] Lopes CT (2010). Cytoscape Web: an interactive web-based network browser. Bioinformatics.

[13] Herschkowitz JI (2007). Identification of conserved gene expression features between murine mammary carcinoma models and human breast tumors. Genome Biol..

[14] Goswami MT (2016). Regulation of complement-dependent cytotoxicity by TGF-beta-induced epithelial-mesenchymal transition. Oncogene.

[15] Mackiewicz A (1990). Transforming growth factor beta 1 regulates production of acute-phase proteins. Proc. Natl. Acad. Sci. USA.

[16] Mishra R (2008). AMP-activated protein kinase inhibits transforming growth factor-beta-induced Smad3-dependent transcription and myofibroblast transdifferentiation. J. Biol. Chem..

[17] Saito A (2013). An integrated expression profiling reveals target genes of TGF-beta and TNF-alpha possibly mediated by microRNAs in lung cancer cells. PLoS ONE.

[18] Shirakihara T (2011). TGF-beta regulates isoform switching of FGF receptors and epithelial-mesenchymal transition. EMBO J..

[19] Zhang D (2000). TAK1 is activated in the myocardium after pressure overload and is sufficient to provoke heart failure in transgenic mice. Nat. Med..

[20] Ju W (2009). Renal gene and protein expression signatures for prediction of kidney disease progression. Am. J. Pathol..

[21] Curtis C (2012). The genomic and transcriptomic architecture of 2,000 breast tumours reveals novel subgroups. Nature.

[22] Padua D (2008). TGFbeta primes breast tumors for lung metastasis seeding through angiopoietin-like 4. Cell.

[23] Barabasi AL, Gulbahce N, Loscalzo J (2011). Network medicine: a network-based approach to human disease. Nat. Rev. Genet..

[24] Venkatesan K (2009). An empirical framework for binary interactome mapping. Nat. Methods.

[25] Kang Y, Chen CR, Massague J (2003). A self-enabling TGFbeta response coupled to stress signaling: Smad engages stress response factor ATF3 for Id1 repression in epithelial cells. Mol. Cell.

[26] Albert R, Jeong H, Barabasi AL (2000). Error and attack tolerance of complex networks. Nature.

[27] Kresse SH (2009). LSAMP, a novel candidate tumor suppressor gene in human osteosarcomas, identified by array comparative genomic hybridization. Genes Chromosomes Cancer.

[28] Chen J (2003). The t(1;3) breakpoint-spanning genes LSAMP and NORE1 are involved in clear cell renal cell carcinomas. Cancer Cell.

[29] Taguchi A (2015). MAPRE1 as a plasma biomarker for early-stage colorectal cancer and adenomas. Cancer Prev. Res. (Phila.).

[30] Gurski LA (2015). Relocation of CLIC1 promotes tumor cell invasion and colonization of fibrin. Mol. Cancer Res..

[31] Faca VM (2008). Proteomic analysis of ovarian cancer cells reveals dynamic processes of protein secretion and shedding of extra-cellular domains. PLoS ONE.

[32] Cao LL (2018). SERPINA3 silencing inhibits the migration, invasion, and liver metastasis of colon cancer cells. Dig. Dis. Sci..

[33] Zhang Z (2017). SRGN-TGF beta 2 regulatory loop confers invasion and metastasis in triple-negative breast cancer. Oncogenesis.

