# Supplementary material for: A plasma protein derived TGFβ signature is a prognostic indicator in triple negative breast cancer
Source: NPJ Precis Oncol. 2019 Apr 2;3:10. doi: 10.1038/s41698-019-0082-5 (PMC6445093; doi:10.1038/s41698-019-0082-5)
Supplement: Supplementary file 2 — Supplementary Figures [file 41698_2019_82_MOESM2_ESM.pptx]

## Slide 1
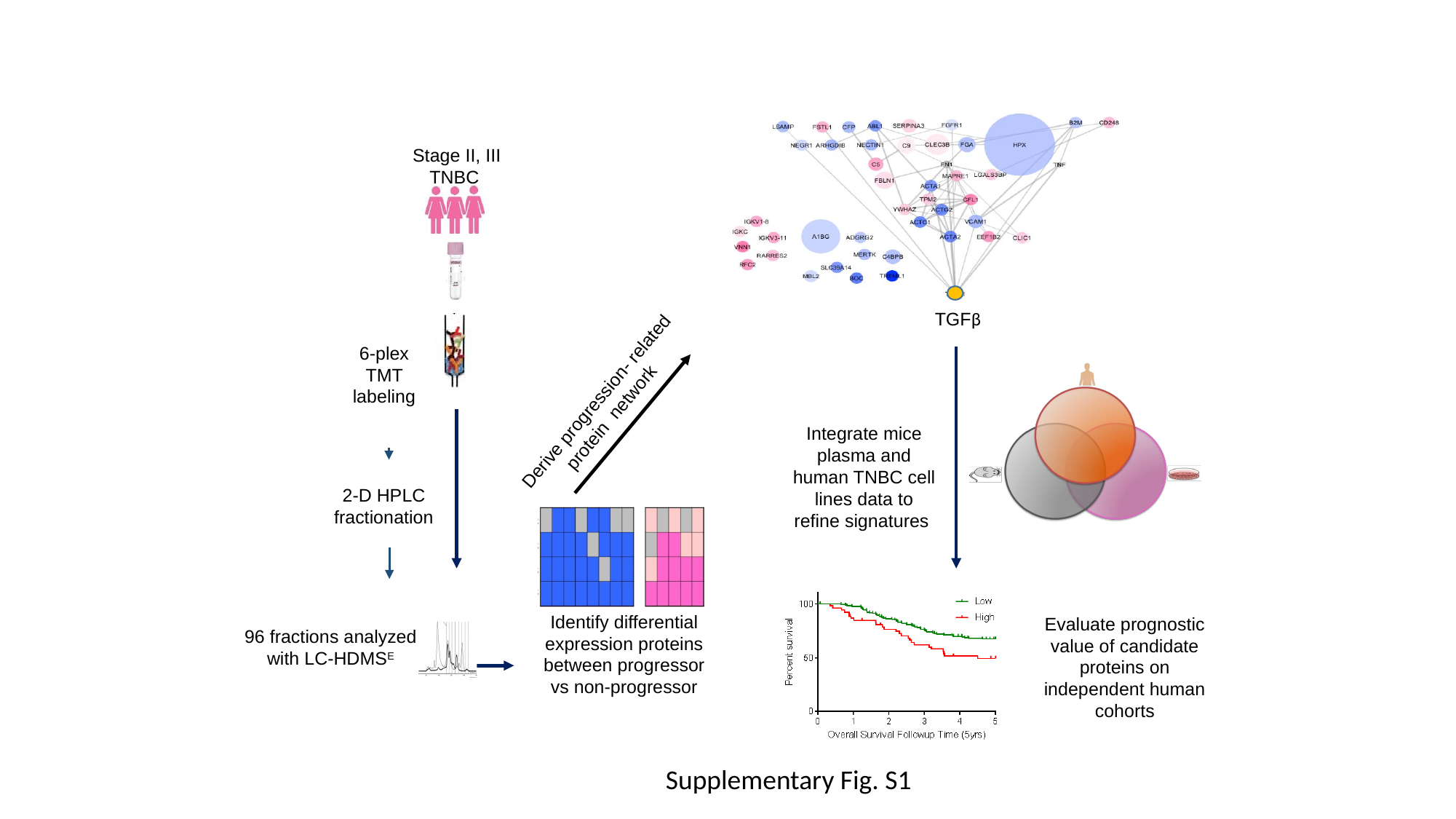

Stage II, III TNBC
TGFβ
6-plex TMT labeling
Derive progression- related protein network
Integrate mice plasma and human TNBC cell lines data to refine signatures
2-D HPLC fractionation
Identify differential expression proteins between progressor vs non-progressor
Evaluate prognostic value of candidate proteins on independent human cohorts
96 fractions analyzed with LC-HDMSE
Supplementary Fig. S1

## Slide 2
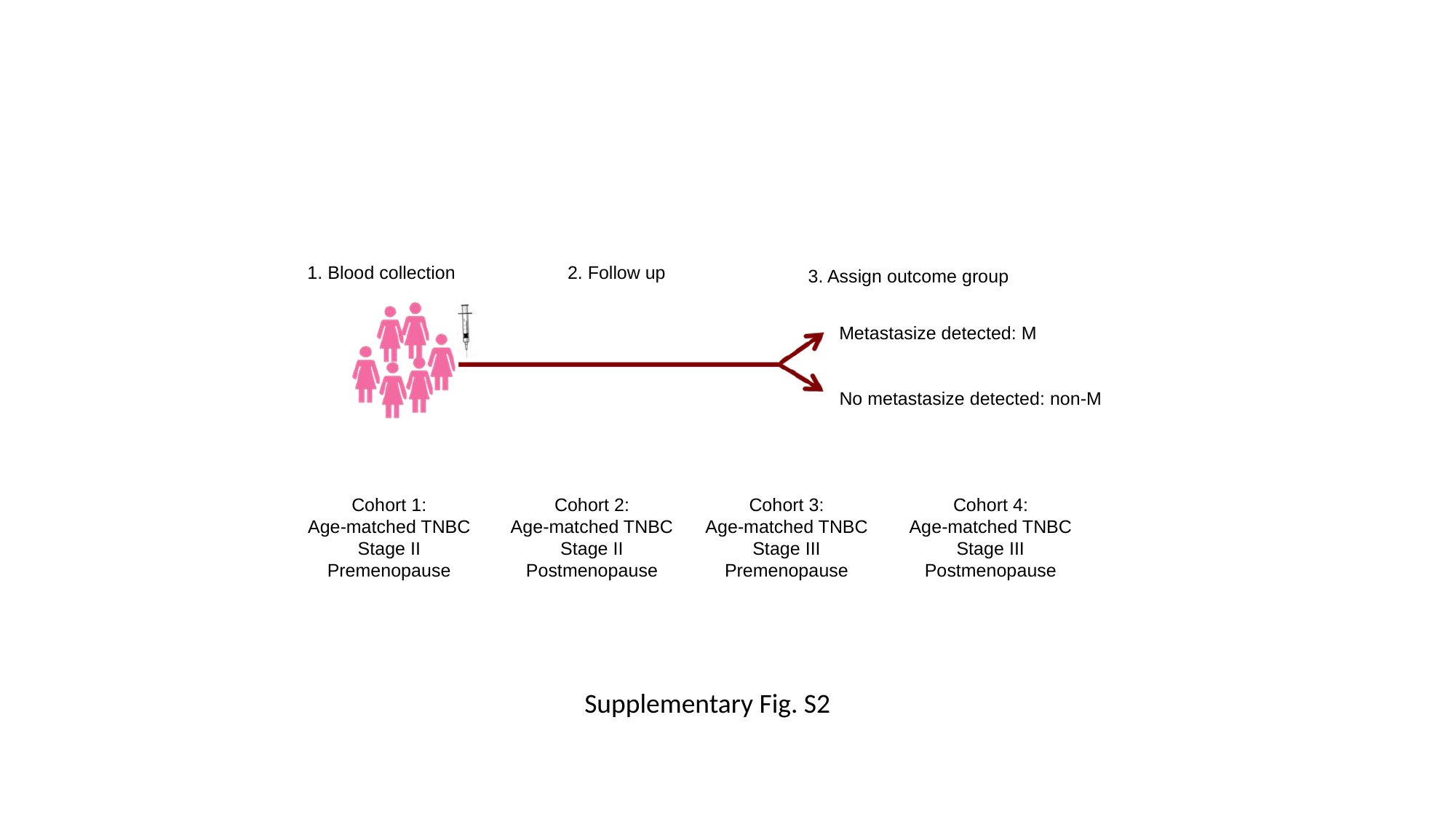

2. Follow up
1. Blood collection
3. Assign outcome group
Metastasize detected: M
No metastasize detected: non-M
Cohort 1:
Age-matched TNBC
Stage II
Premenopause
Cohort 2:
Age-matched TNBC
Stage II
Postmenopause
Cohort 3:
Age-matched TNBC
Stage III
Premenopause
Cohort 4:
Age-matched TNBC
Stage III
Postmenopause
Supplementary Fig. S2

## Slide 3
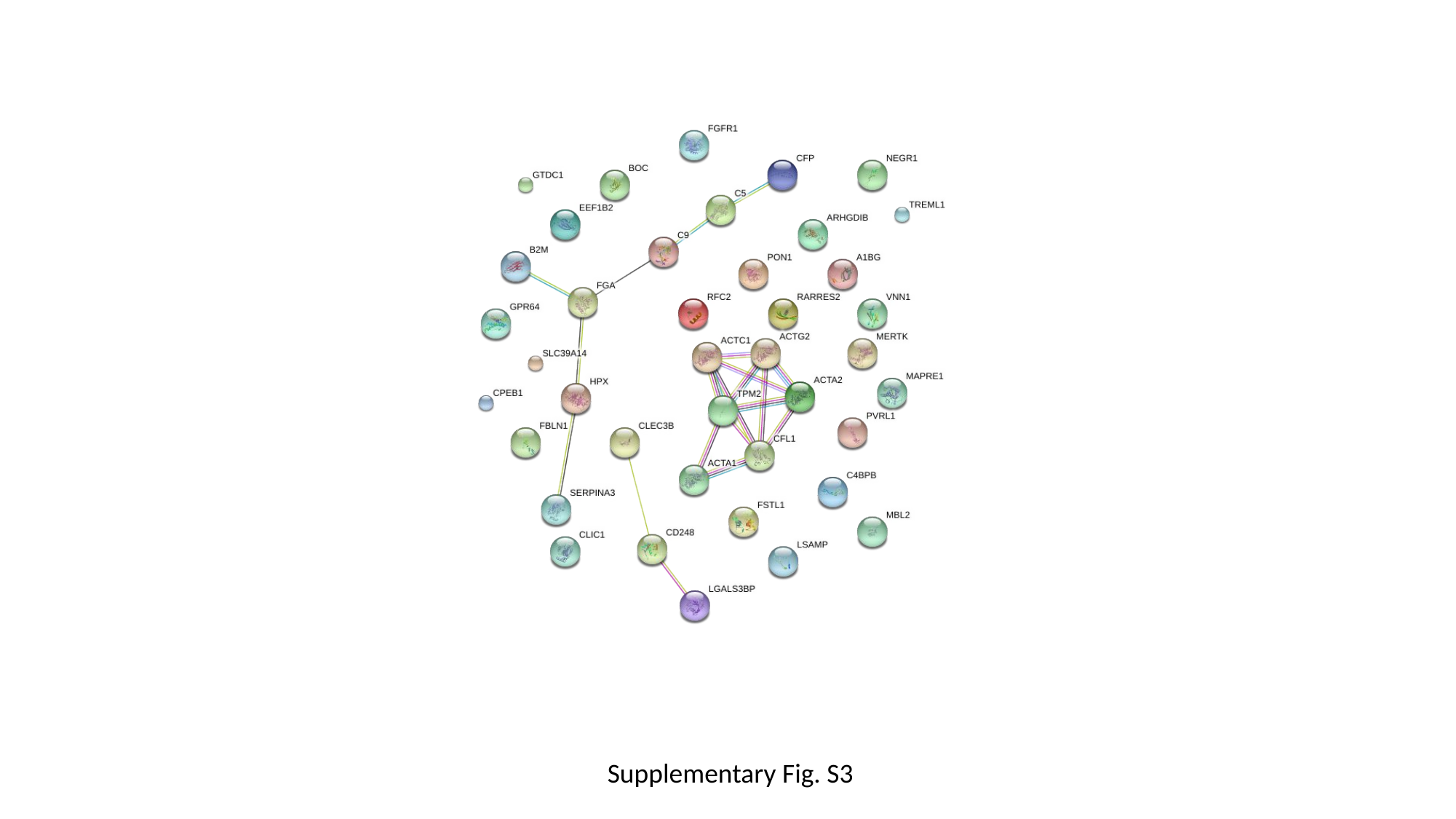

Supplementary Fig. S3

## Slide 4
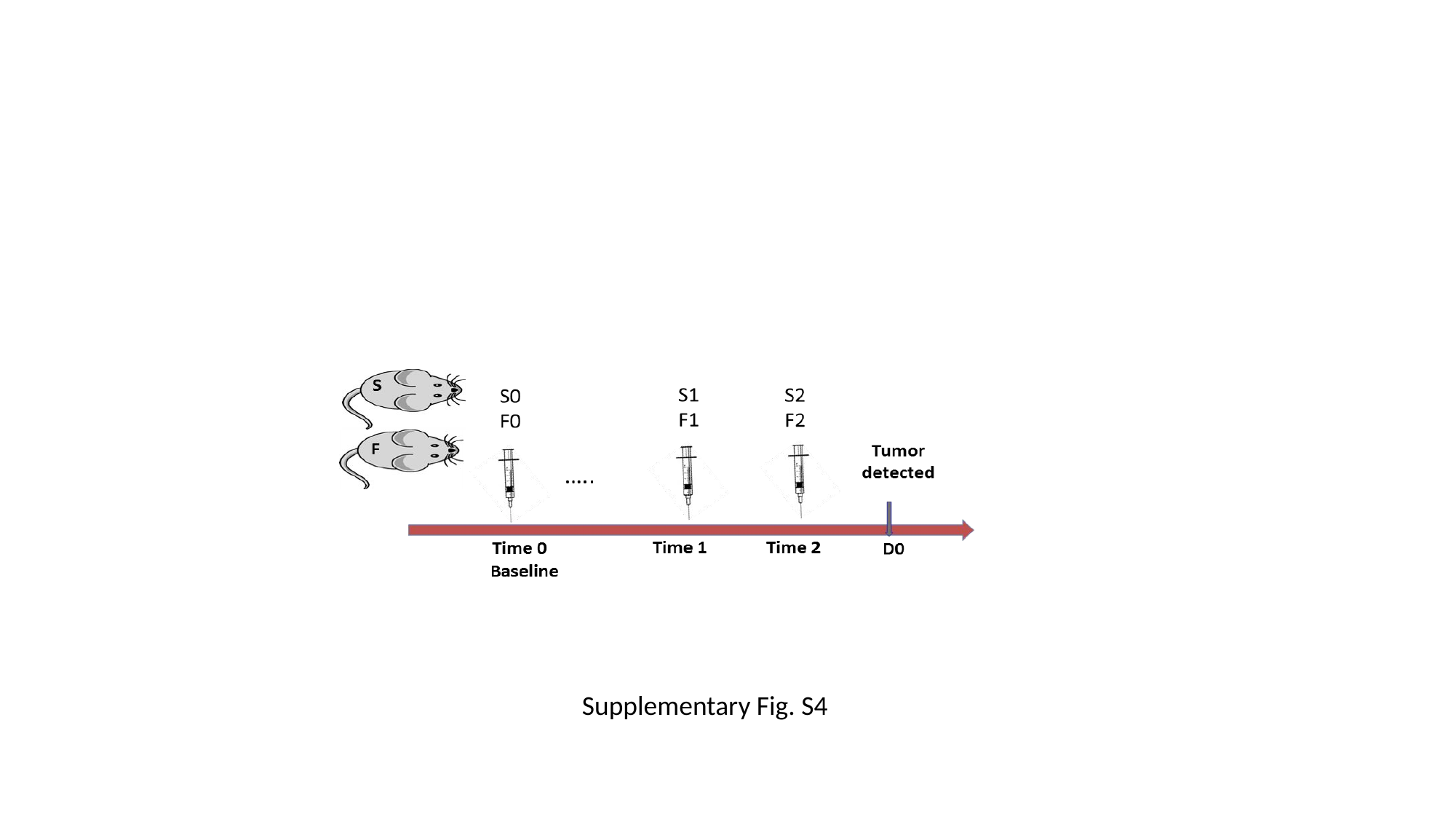

Supplementary Fig. S4

## Slide 5
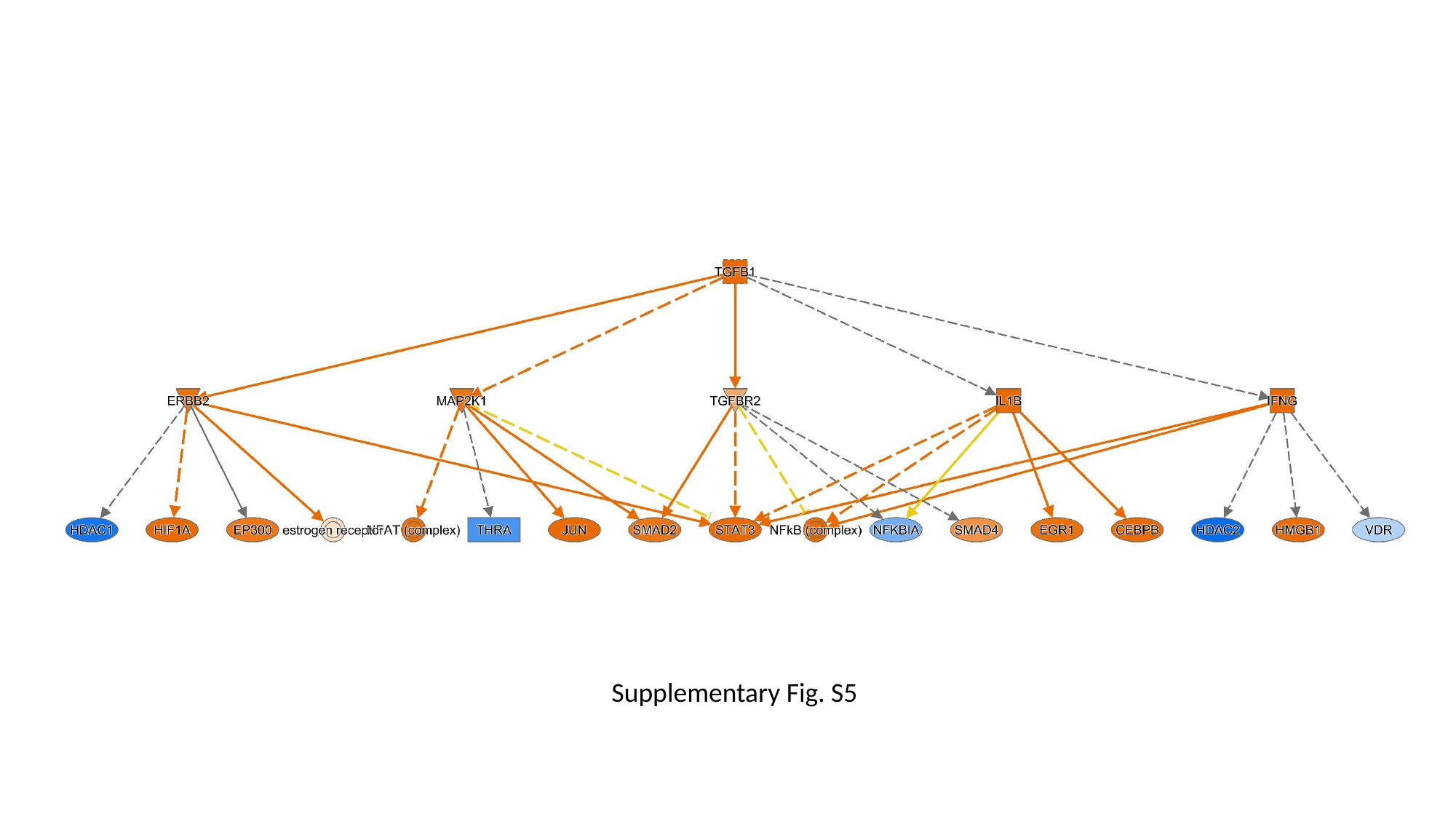

Supplementary Fig. S5

## Slide 6
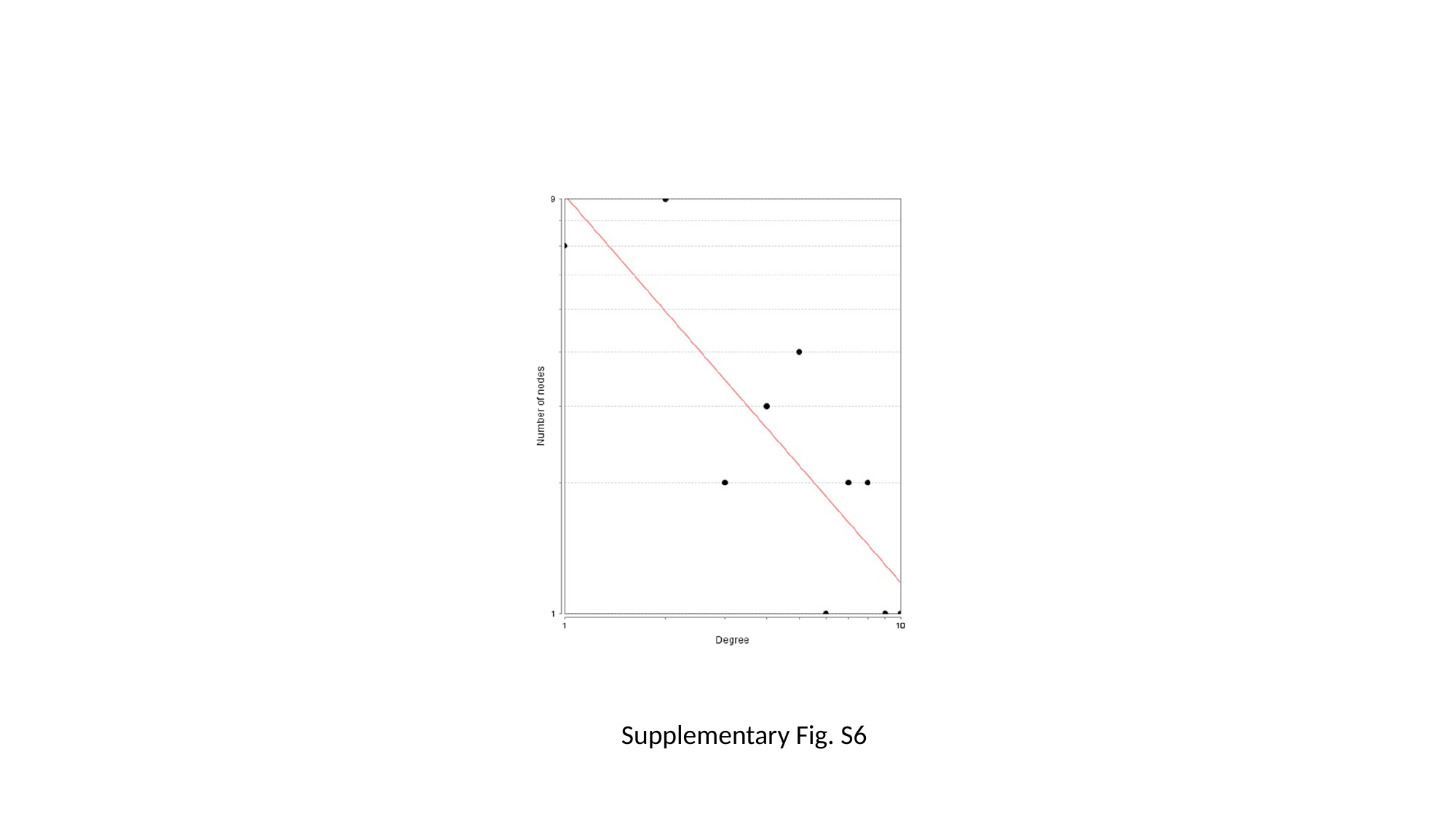

Supplementary Fig. S6

## Slide 7
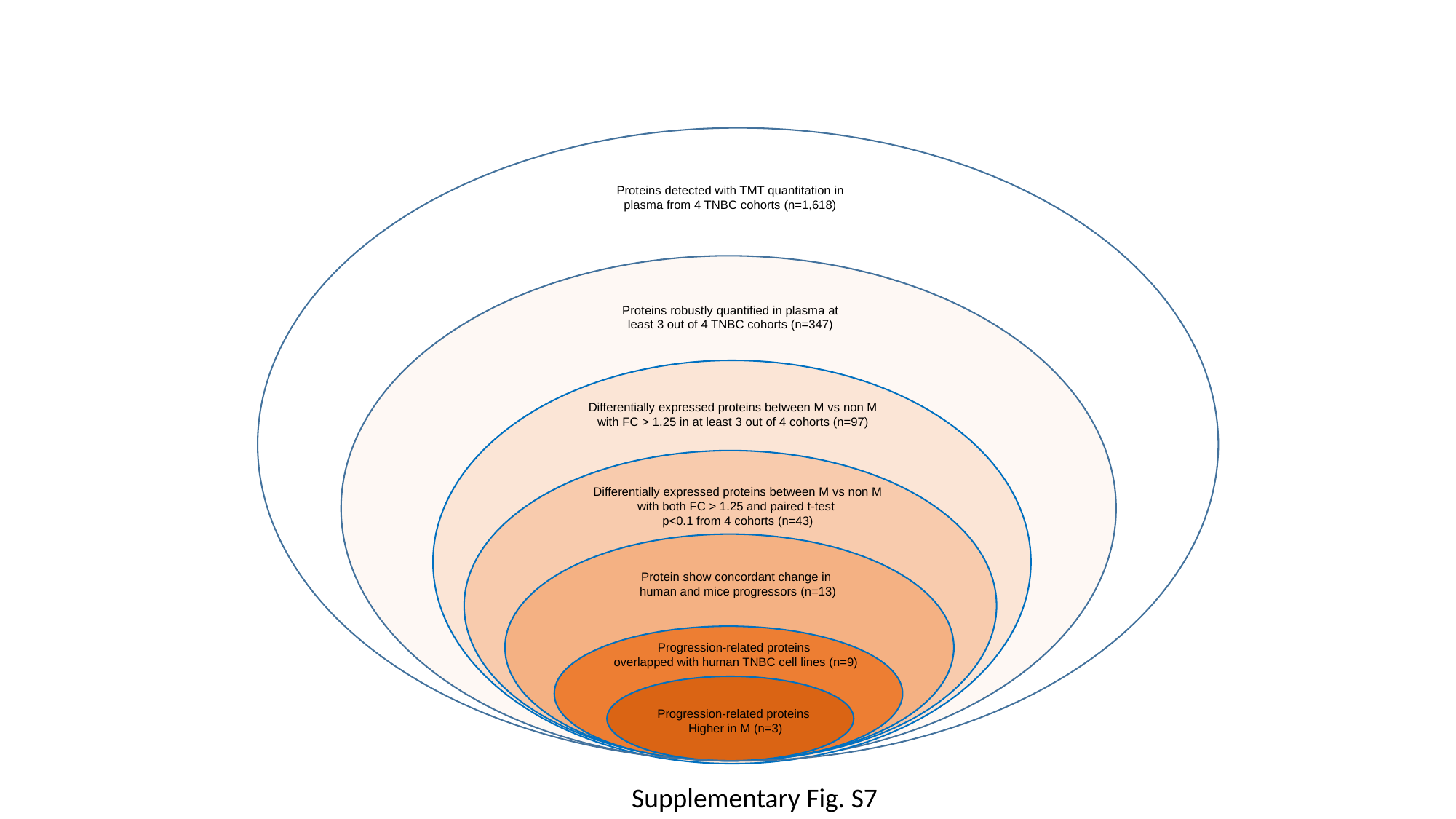

Proteins detected with TMT quantitation in
plasma from 4 TNBC cohorts (n=1,618)
Proteins robustly quantified in plasma at
least 3 out of 4 TNBC cohorts (n=347)
Differentially expressed proteins between M vs non M
with FC > 1.25 in at least 3 out of 4 cohorts (n=97)
Differentially expressed proteins between M vs non M
with both FC > 1.25 and paired t-test
p<0.1 from 4 cohorts (n=43)
Protein show concordant change in
human and mice progressors (n=13)
Progression-related proteins
overlapped with human TNBC cell lines (n=9)
Progression-related proteins
Higher in M (n=3)
Supplementary Fig. S7
